# Enzootic frog pathogen *Batrachochytrium dendrobatidis* in Asian tropics reveals high ITS haplotype diversity and low prevalence

**DOI:** 10.1038/s41598-018-28304-1

**Published:** 2018-07-04

**Authors:** Milind C. Mutnale, Sachin Anand, Lilly M. Eluvathingal, Jayanta K. Roy, Gundlapally S. Reddy, Karthikeyan Vasudevan

**Affiliations:** 1 CSIR-Centre for Cellular and Molecular Biology, Laboratory for the Conservation of Endangered Species, Hyderabad, Telangana India; 2 Biology Department, Occidental College, Los Angeles, California 90041 USA; 3 Department of Life Science and Bioinformatics, Assam University, Diphu Campus, Karbi Anglong, Assam 782460 India

## Abstract

Emerging Infectious Diseases (EIDs) are a major threat to wildlife and a key player in the declining amphibian populations worldwide. One such EID is chytridiomycosis caused by *Batrachochytrium dendrobatidis* (*Bd*), a fungal pathogen. Aetiology of *Bd* infection is poorly known from tropical frogs in Asian biodiversity hotspots. Surveys were carried out in four biodiversity hotspots to ascertain the status of *Bd* fungus. We collected a total of 1870 swab samples from frogs representing 32 genera and 111 species. Nested PCRs revealed low prevalence (8.4%) and high *Bd* haplotype richness was revealed after sequencing. We document 57 *Bd* Internal Transcribed Spacer region (ITS) haplotypes, of which 46 were unique to the global database. *Bd* ITS region showed indels at the Taqman binding site and qPCR reverse primer binding site, suggesting qPCR is unsuitable for diagnosis in Asian *Bd* coldspots. Our median-joining network and Bayesian tree analyses reveal that the Asian haplotypes, with the exception of Korea, formed a separate clade along with pandemic *Bd*GPL (*Bd* Global Panzootic Lineage) haplotype. We hypothesise that the frog populations in Asian tropics might harbour several endemic strains of *Bd*, and the high levels of diversity and uniqueness of *Bd* haplotypes in the region, probably resulted from historical host-pathogen co-evolution.

## Introduction

Chytridiomycosis, an Emerging Infectious Disease (EID) is responsible for declines or extinctions of over 200 amphibian species^1^ and nearly 700 amphibian species are affected by it worldwide^2^. It is caused by aquatic fungal pathogens, *Batrachochytrium dendrobatidis* (*Bd*) and *Batrachochytrium salamandrivorans* (*Bsal*). *Bd* is known to cause infections in anurans^3^, salamanders^3^, and caecilians^4^ while *Bsal* is reportedly salamander^5^ specific. However, a recent study confirms *Bsal* infection on anurans^6^. *Bd* causes hyperkeratosis of the epidermal layer on the skin of frogs, disrupting osmoregulation, exchange of ions, and eventually causing cardiac arrest leading to death^7^. With the exceptions of Asia and Antarctica, mass mortality has been observed on all continents. Measuring prevalence and culturing of *Bd* in different geographic regions have advanced our understanding of the aetiology of the disease. In the last decade, as reports on the prevalence of *Bd* emerged, it became evident that frog populations in Asia have low *Bd* prevalence^8–14^; such regions are referred to as coldspots for *Bd* infection^15^. Four of the five known *Bd* lineages are thought to be endemic and enzootic^16–19^. As a consequence, virulence of different *Bd* strains^20^ and the immune responses of frogs vary^21^. The Global Panzootic Lineage or *Bd*GPL^16^, is the pathogenic lineage responsible for causing mortality of frogs globally.

There were many hypothesis about the origin and spread of *Bd*. Out-of-Africa hypothesis was based on the detection of *Bd* in historical collections of the African clawed frog, *Xenopus laevis*, dating back to 1938^22^. Since *X.laevis* did not succumb to *Bd*, and it was exported to many countries for experimentation^23^ it might have led to the spread of *Bd*^24^. Later, Goka *et al*.^8^ proposed ‘*Bd* out-of-Asia’ hypothesis after detecting *Bd* in *Andrias japonicas*, collected in 1902. New hypotheses are in contention after *Bd* was detected in a historical collection of frogs (*Acris blanchardi* and *Rana sphenocephala*) from 1888 in the USA^25^, and (*Hypsiboas pulchellus*) in Brazil from 1894^19^. A recent global study conducted using the next generation sequencing and phylogenetic analysis of 234 isolates revealed the *Bd* origin to be Asia^26^. Tip dating analysis predicts *Bd*GPL might have originated 120–50 years ago^26^. Clearly, *Bd* infections are new to frog populations globally, and the factors that lead to their susceptibility to infections are still poorly understood.

The status of chytridiomycosis in Asia is poorly understood, with the exception of a large scale survey involving 15 countries in Asia, that revealed low *Bd* prevalence, and predicted the widespread occurrence of *Bd* in several parts of Asia^9^. The first report of a *Bd* outbreak came from Japan in 2006^27^. Following this, intensive surveys revealed several haplotypes of *Bd* in Japan^8^, China^28^, and Korea^18^. Such areas are referred to as coldspots of *Bd* infection and are typically characterised by a high number of haplotypes and low prevalence^15^. From the global *Bd* phylogeny, it is evident that, Korea harbors two enzootic *Bd* lineages namely *Bd*ASIA-1 and *Bd*ASIA-2/*Bd*BRZIL^26^.

As reports on *Bd* from Asia are growing, it has widened the scope of investigations on the role of the pathogenic strain in the region, and the measures to be taken to secure several narrowly endemic frog populations from lethal infections. India lies at the intersection of four globally recognised megabiodiversity hotspots^29^ with high levels of frog species richness and endemism. *Bd* is expected to be widespread in these hotspots^9,30–32^. India has over 375 frog species, and the list is growing (see, http://amphibiaweb.org/). With such high frog species richness, the stakes are high for understanding the role of *Bd* on frog populations in the region. Apart from a few scattered efforts in the Western Ghats of India^10,33–35^, the status of *Bd* in the hotspots is unknown. Since chytridiomycosis is a notifiable aquatic disease, and country-wide reports are far from complete, there is a compelling case for countries like India to report the status of *Bd*. With these considerations, we measured the prevalence of *Bd* in frog populations from four biodiversity hotspots in India, using nested Polymerase Chain Reaction (PCR). We found that *Bd* is prevalent at a low level in all the hotspots in India. We retrieved several *Bd* haplotypes by targeting the ribosomal Internal Transcribed Spacer region (ITS) and several of them were unique and therefore, we predict that there are enzootic lineages yet to be discovered from the region. Our haplotype network revealed that enzootic haplotypes clustered around *Bd*GPL. The haplotype network further lends support to the hypothesis that *Bd* might have originated from Asia. We investigated the efficiency of conventional assays used for detection of *Bd*, and emphasise the need for efficient assays for detecting *Bd* in Asian coldspots. These findings have important implications for the ongoing global effort to understand impacts of the disease.

## Results

### *Bd* detection and prevalence

A total of 1870 swab samples (147 locations) from frogs that belonged to 32 genera and 111 species were analysed. Of these, 158 samples showed amplification in the nested PCR method for *Bd* (Table 1). We initially analysed 1050 samples using both qPCR and Nested PCR methods. Both methods varied in their sensitivity; qPCR and nested PCR showed 33 and 119 positive samples, respectively. Both qPCR and nested PCR methods had only 19 positives in common. Since nested PCR showed greater sensitivity in detecting *Bd*, it was used as our primary assay for measuring prevalence.

**Table 1:** *Bd* prevalence in the different geographic regions in India from 2012 to 2017.

Region	Location	No. of samples	No. of Species	Nested PCR positive	Prevalence (95% CI)	Number of haplotypes
Andaman and	Little Andaman	42	13	9	10.5% (8–13%)	25
Nicobar Islands (AN)	North Andaman	36		0		
	Middle Andaman	168		27		
	South Andaman	134		9		
	Car Nicobar	58		11		
	Great Nicobar	140		5		
North East Hills (NE)	Imphal	133	2	0	0% (0–1%)	0
	Ukurul	67		0		
Eastern Himalaya (EH)	Darjeeling	13	30	1	3.9% (2–7%)	
	Dibang valley	140		6		3
	Eagle Nest wildlife sanctuary	26		0		
Western Himalaya (WH)	Corbett	28	8	6	14% (7–26%)	8
	Nainital	7		1		
	Dehradun	4		0		
	Dhanaulti	11		0		
Eastern Ghats (EG)	Araku	90	2	2	2.2% (0.6–7%)	Unidentified
Western Ghats (WG)	Munnar	245	63	27	10.4% (8–12%)	
	Kalakad Mundanthurai Tiger Reserve	158		32		36
	Srivilliputhur Wildlife Sanctuary	49		7		
	Taleigao	16		6		
	Dharwad	35		5		
	Sirsi	21		1		
	Sakleshpur	71		0		
	Kolhapur	2		0		
	Khireshwar	17		1		
	Tilari	139		2		
	Matheran	20		0		
**Total**		**1870**		**158**	**8.4% (7–9%)**	

Prevalence was the highest in Micrixalidae with 17.4% (95% CI 7–37%) (Table 2); all frogs in this family are endemic to the Western Ghats. Overall, the prevalence of chytridiomycosis in frog populations that were sampled in different regions of India was 8.44% (95% CI 7–97%). Seasonal variation in *Bd* prevalence coincided with the onset of the monsoons. *Bd* prevalence gradually increased from June, peaked in July (23.4%), and gradually dropped in September. From this point the prevalence progressed to 14.2% in October and subsequently decreased from November onwards (Table 3).

**Table 2:** Prevalence of *Bd* in frog families with the 95% confidence interval.

Family	Positive	Samples	Prevalence	Lower limit (95% CI)	Upper limit (95% CI)
Bufonidae	14	165	8.5%	5.1	13.7
Dicroglossidae	73	971	7.5%	6	9.3
Megophryidae	0	1	0%	0	94.9
Micrixalidae	4	23	17.4%	7.0	37.1
Microhylidae	13	89	14.6%	8.7	23.4
Nyctibatrachidae	10	100	10%	5.5	17.4
Ranidae	9	145	6.2%	3.3	11.4
Ranixalidae	8	66	12.1%	6.3	22.1
Rhacophoridae	27	309	8.7%	6.1	12.4
Unidentified anurans	0	1	0%	0	94.9
**Total**	**158**	**1870**	**8.4%**	**7**	**9**

**Table 3:** Number of samples collected during the year, with number of *Bd* positive samples indicated in brackets, showing seasonal variation in *Bd* prevalence.

Month	2012	2013	2014	2015	2016	2017	Total	Prevalence (95% CI)
Jan	0	0	0	172(33)	0	248(16)	420(49)	**11.6% (8.9–15%)**
Feb	0	0	0	54(5)	0	140	194(5)	**2.57%** (**1.1–5.8%)**
Mar	0	21	0	62(4)	0	40	123(4)	**3.2% (1.2–8%)**
Apr	0	15(2)	0	12	15	0	42(2)	**4.7% (1.3–15.7%)**
May	0	14(3)	23(5)	20	120	0	177(8)	**4.5% (2.3–8.6%)**
Jun	0	16(1)	12(1)	125(10)	30	0	183(12)	**6.5% (3.7–11.1%)**
Jul	0	0	0	140(35)	9	0	149(35)	**23.4% (17.4–30.9%)**
Aug	0	0	18(6)	41(7)	149(2)	0	208(15)	**7.2% (4.4–11.5%)**
Sep	0	0	0	17(1)	0	0	17(1)	**5.8% (0.3–26.9%)**
Oct	0	0	16(4)	12	0	0	28(4)	**14.2% (5.6–31.4%)**
Nov	29(5)	0	30(2)	39(5)	1	0	99(12)	**12.1% (7–20%)**
Dec	0	0	66(2)	68(7)	96(2)	0	230(11)	**4.7% (2.6–8.3%)**

**Figure 1:**
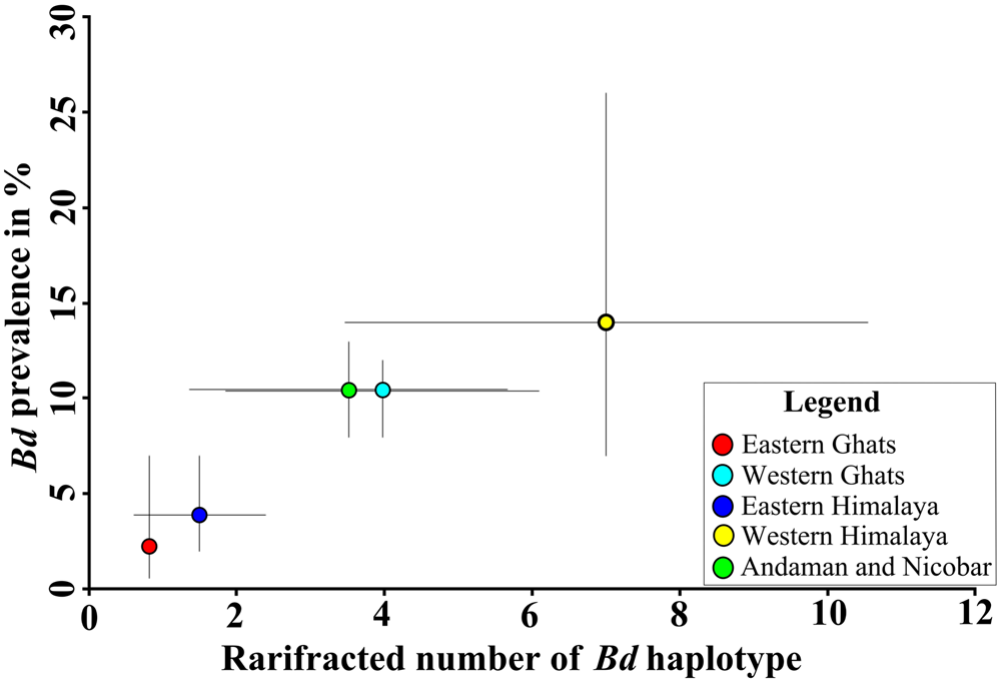
Graph representing the prevalence on Y axis and rarifracted number of *Bd* haplotypes on X axis with colour codes indicating different geographic regions. Error bars represent the 95% confidence interval upper and lower limits.

There was no significant difference in *Bd* prevalence among frog populations in various geographic regions (F value = 0.51, df = 5, p = 0.766). Rarefaction revealed that the Western Himalaya region had the maximum number of haplotypes of *Bd* (Fig. 1). Large variations in both prevalence and haplotypes richness were observed within the different geographic regions (Fig. 1).

### ITS haplotype diversity and network

**Figure 2:**
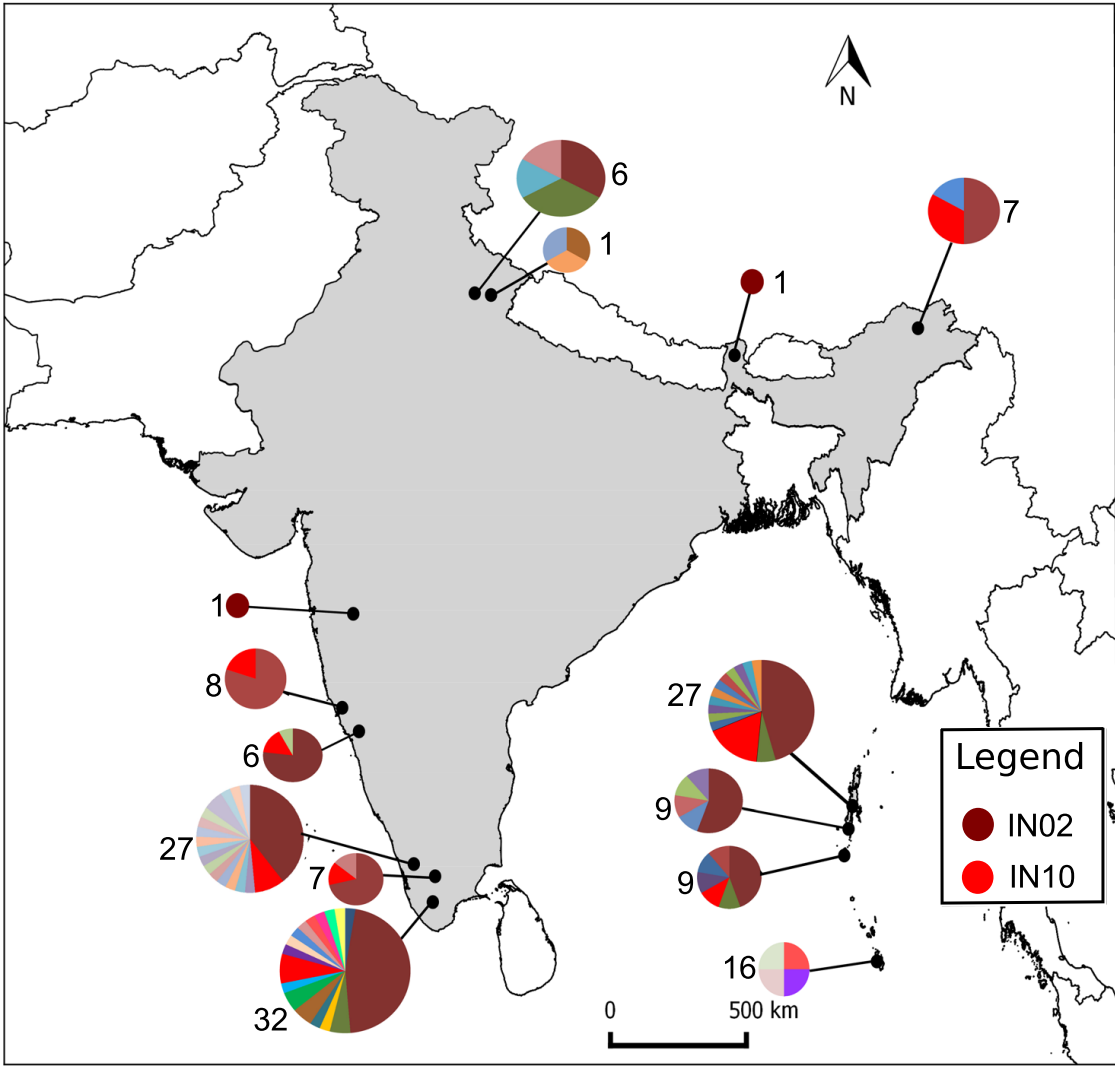
*Bd* haplotypes recovered from the different locations. Brown coloured haplotype IN02 is wide spread, except in the Nicobar Islands. Red coloured haplotype IN10 is present at all the locations sampled except in the Eastern Himalayas. *Bd *haplotype richness was highest in the Western Ghats. Numbers adjacent to the pie charts represent number of positive *Bd* samples. A detailed summary of the percentage of each haplotype of *Bd* at different locations is provided in Supplementary Table S4. [Maps were prepared using QGIS 2.10 (https://www.qgis.org/en/site/) and were further modified using Inkscape 0.91 (https://inkscape.org/en/)].

From 158 *Bd* positive samples, 57 haplotypes were retrieved. We found single haplotypes in all samples, except for 31 samples, wherein multiple haplotypes were detected. From the positive samples, 49 sequences consisting of 34 haplotypes were resolved through cloning. Among them, 46 haplotypes were unique to India, and 11 haplotypes matched with those reported from China, Japan, South Africa, USA, and Italy. Out of the 57 haplotypes, 33 were exclusively from mainland India, and 19 were from Andaman and Nicobar Islands. Only five haplotypes were shared between the mainland and the Islands. Haplotypes varied in their amplicon size ranging from 247 to 263 bp. Two haplotypes (IN02 and IN10) contributed to 65% of the *Bd* positive samples. Haplotype IN02 was the most common one, and it was represented in 51% of the *Bd* positive samples. Haplotype IN10 was present in 20 samples, from all regions that had *Bd*, except for the Western Himalayas and the Nicobar Islands (Fig. 2). We estimate that there were 147 haplotypes (SE ± 0.44) involved in our samples after accounting for the undetected ones.

Four haplotypes, IN05, IN17, IN31, and IN55 had mutations at the Taqman probe binding site. They contributed to 3.1% (N = 158) of positives in our sample. Two out of the four haplotypes showed an addition of two adenine residues at the Taqman binding site and the other haplotypes showed transition and transversion of a single nucleotide base (Supplementary Fig. S1).

In the Bayesian tree, haplotypes from Korea, Japan, and Brazil formed a distinct clade whereasthe haplotypes from India grouped with haplotypes from China, Japan, Italy, South Africa, and Texas. Both the clades were supported by posterior probability values. As expected, the Taqman binding mutation haplotypes, IN05, IN55, China CN 30, Japan JP02, JP09, and JP10 formed a separate clade (Supplementary Fig. S2).

**Figure 3:**
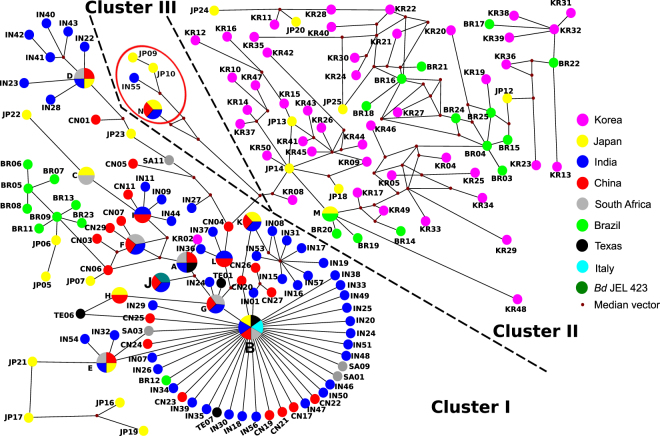
Median-joining haplotype network of *Bd* ITS region, with coloured circles representing different countries; red cubes represent median vectors (missing haplotype). Length between the two haplotypes is proportional to mutation steps between them. Haplotypes within the red circle have mutations at the Taqman binding site. Due to space limitation, we have not shown all mutation steps in the figure. Abbreviations: A = TE05 SA04 IN13 CN28, B = TE02 IT01 SA08 IN02 CN18 JP01, C = SA10 JP08, D = SA07 IN10 JP11 CN2, E = SA06 JP26 IN03 CN9, F = SA05 IN52 CN8, G = SA02 IN04 CN15, H = JP03 CN14, I = IN45 CN10, J = IN14 *Bd* JEL 423 CN13, K = IN12 CN12 JP04, L = IN06 CN16, M = JP15 BR10, and N = JP02 CN30 IN05. Network 5.0 (http://www.fluxus-engineering.com) was used to create haplotype map and modified using Inkscpae 0.91(https://inkscape.org/en/).

Median-joining Network has three major clusters, the first one, representing a majority of the haplotypes from India, along with China, Japan, Italy, South Africa, and Texas. The second cluster consists of haplotypes mainly from Korea, Japan, and Brazil. The third cluster consists of haplotypes with mutations at the Taqman binding site from India, China, and Japan (Fig. 3). Some haplotypes show no specific association, such as those from Japan, as they are represented in all three clusters. The most common haplotype (IN02) found in India clustered with haplotypes from Italy, South Africa, Texas, China, and Japan (cluster I in Fig. 3). *Bd* JEL 423 haplotype clustered with the haplotypes, IN14 and CN13, from India and China, respectively.

## Discussion

Using samples collected over a period of six years, representing approximately 25% of India’s total frog species, we document for the first time that *Bd* is found in frog populations across India, in the Western Ghats, Eastern Ghats, Himalayas, North East Hills, and Andaman and Nicobar Islands. These geographic regions were predicted as areas that might harbour *Bd*, albeit, with low prevalence^9^. We demonstrate that nested PCR is a sensitive assay to quantify *Bd* prevalence in large samples from *Bd* coldspots that characteristically show low prevalence and a high number of enzootic haplotypes. Nested PCR is less expensive than qPCR^36^, and it has the ability to detect *Bd* haplotypes that might go undetected in qPCR. It provides nested PCR with an unassailable advantage for long-term surveillance in *Bd* coldspots. There is a low prevalence of the *Bd* infection in the Western Ghats, as documented previously^10,34,35^, but this was a common pattern, and it is repeated in other regions, with no significant differences between them. We attribute the overall low prevalence to detection bias, and patchy occurrence (Table 1). The factors leading to patchiness in *Bd* occurrence in relatively homogeneous habitats needs further investigation. Nested PCR fared better than qPCR in retrieving *Bd* from samples, but several rare haplotypes in the samples represented by very few zoospores would have certainly remained undetected. Therefore, the *Bd* prevalence measured by this method would be an underestimation. The magnitude of the bias is unknown and therefore, correction factors elude us at this point. We emphasise the need for sensitive and universal assays for detecting all known *Bd* haplotypes. This would be an important pre-requisite for understanding the dynamics of the disease in *Bd* coldspots. The family with the highest number of *Bd* positive samples, Micrixalidae, is known for species that inhabit fast moving streams (torrent frogs), which is not surprising as the *Bd* zoospores are known to be aquatic. Seasonal variation in abiotic factors such as air temperature and rainfall are known to influence *Bd* prevalence^37–40^. The regions sampled receive two monsoons, viz: the southwest, and the northeast. A sharp rise in prevalence was seen in July and October, and these coincide with the monsoons. Such patterns in annual variation in *Bd* prevalence are known from other parts of the world^37,38,41^.

The number of haplotypes of *Bd* involved in infecting a frog population seems to fluctuate with the prevalence in that population (Fig. 1). This pattern could emerge when a pool of *Bd* haplotypes with varying abundance assort themselves to susceptible hosts, based on haplotype-specific colonisation. It points at the hitherto undervalued role of *Bd* haplotypes in the persistence of infections on frogs. Future work obtaining cultures of *Bd*, from coldspots would be an important step towards revealing whether the outcome of enzootic *Bd* strains in frogs is a result of competitive superiority over *Bd*GPL strain or the naivety of frog populations to *Bd*GPL infections^15^. The occurrence of coldspots of *Bd*, within global amphibian biodiversity hotspots, such as the case presented here, should be understood in the context of theoretical expectations of host-pathogen evolution^15^. Long-term monitoring of *Bd* prevalence in these areas would be vital in answering these questions.

ITS region is the fungal DNA-barcode region used for species level identification^42^. Using the nested PCR assay, Bai *et al*.^28^ reported 30 new haplotypes from mainland China, with two widespread haplotypes, CN18 and CN02. The haplotype CN18 was present in 78% of the total number of positive samples from this study. Similarly, we identified a single dominant haplotype, IN02, in 51% of the *Bd* positive samples in India. IN02 was also identical to haplotypes CN18, JP01, SA08 from China, Japan, and South Africa, respectively. We observed similarity in the reports from China and our study, wherein, low *Bd* prevalence was also associated with multiple haplotypes^8,28^. Based on this evidence, coldspots should be investigated for the role of dominant haplotypes involved in infections.

We now know that *Bd *occurs *in-situ* with several enzootic haplotypes in India. In this study, we could sequence only a little over a third of the *Bd* haplotypes that were probably present in the samples. A single strain of *Bd* could have multiple *Bd* ITS regions, resulting in many haplotypes^17^; several endemic strains in Asia could be involved in causing infections.

Global trade in amphibians is held responsible for the spread of *Bd* across the world^43–46^. Historically, India was a major exporter of three large aquatic frogs: *Hoplobatrachus tigerinus*, *Euphlyctis hexadactylus*, and *Hoplobatrachus crassus* until early 1980’s^47,48^. Trade of frogs in India ceased after a ban imposed on the collection and export of frogs^49^. There is no known record of the import of frogs into India. Migratory birds, crayfish, and movement of researchers are some of the possible sources^50–52^ that might have seeded *Bd* fungus into India. The preponderance of enzootic haplotypes, and their close relationship to *Bd*GPL, prompts us to hypothesise that frog populations in India might harbour several endemic strains of *Bd*, and the high levels of diversity and uniqueness of *Bd* haplotypes in the region, is probably resulted due to historical host-pathogen co-evolution.

Worldwide, Taqman based quantitative PCR is used as the primary diagnostic tool for detecting *Bd*^53^. The efficacy of this assay was questioned because mutations were identified at the Taqman binding site^8^. We found 30 haplotypes in the database that have mutations at the Taqman binding site. Three haplotypes from China, 13 from Japan, 9 from Korea, and 5 from Brazil show mutations at the Taqman binding site either as indels or as transition-transversions (Supplementary Table S1 and Fig. S1). We additionally report 15 haplotypes with mutations at the reverse primer binding site (Supplementary Table S2 and Fig. S3). The Taqman probe Chytr MGB was designed using 29 *Bd* sequences from Australia, the USA, Panama, and Ecuador^54^. Twenty six out of the 29 sequences were identical, and the remaining three sequences showed indels^54^. Presently, we have more than 250 ITS1 sequence accessions in NCBI GenBank, obtained either from simple PCRs or Nested PCRs^8,18,28,35,55^. If *Bd* coldspots are indeed associated with high *Bd* haplotype richness, and Taqman based qPCR is used as the primary assay, the probability of false negatives would increase, resulting in the failure to report *Bd*, when it occurs with low prevalence. Multiple sequence alignment of *Bd* ITS1 revealed that there were no conserved sites, making it an unsuitable target for new Taqman probes. Therefore, we speculate that Chytr MGB Taqman probe might not serve as an effective probe for *Bd* detection in Asian coldspots.

*Bd* fungus is present in all the frog hotspots in India and it needs to be matched with a heightened level of surveillance in the region. Our data highlight the need for sensitive and specific assays to detect them as conventional assays show limitations in detecting different haplotypes. Globally, Asia has the highest number of *Bd* haplotypes and the least number of *Bd* related mortalities. This presents an opportunity for investigating the strains involved in infections in the region, and the persistence of enzootic strains in Asian tropical frog populations.

## Methods

### Sample collection

**Figure 4:**
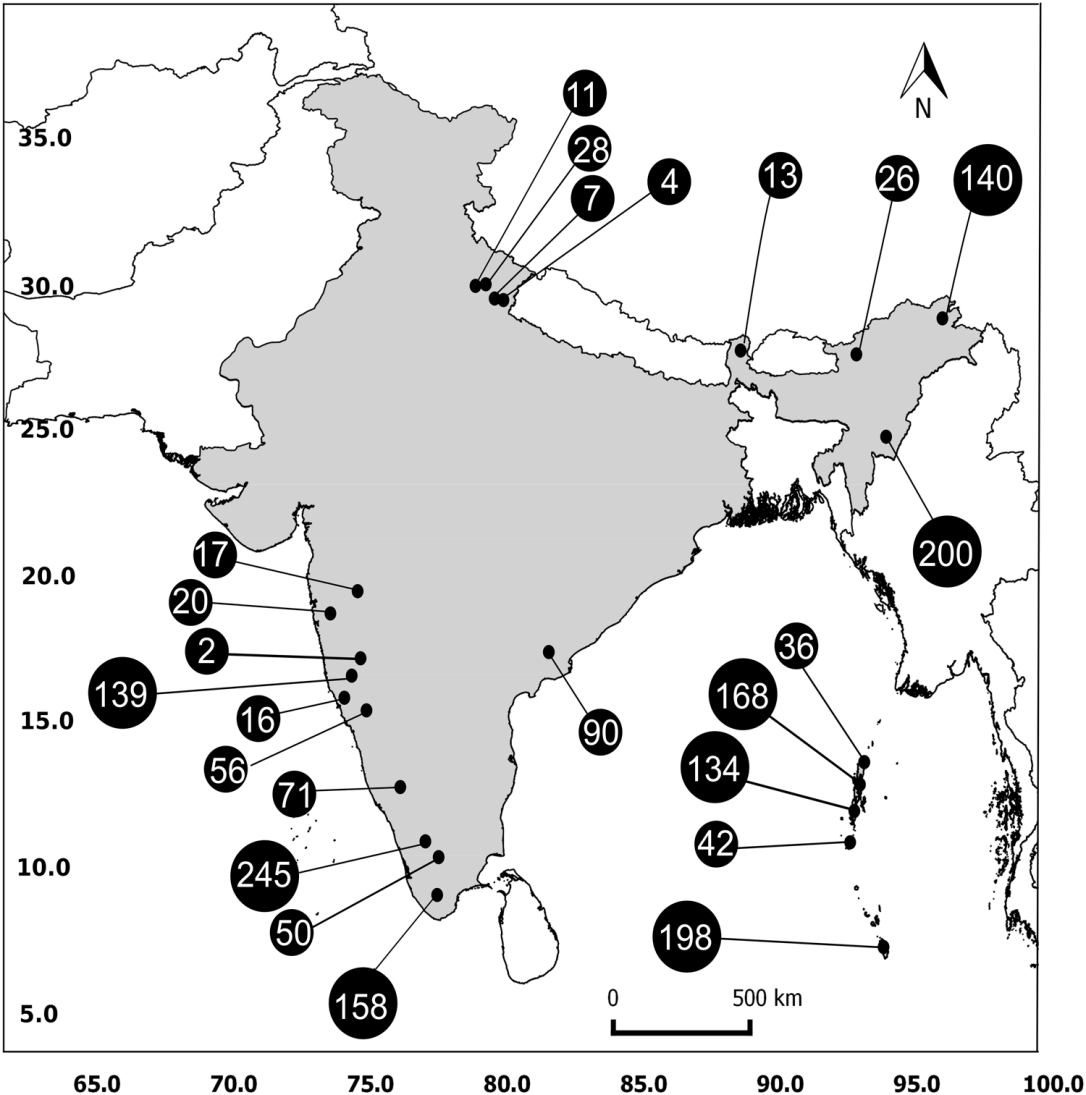
Sampling locations in India (shaded in grey) with numbers indicating the number of swabs collected. [Maps were prepared using QGIS 2.10 (https://www.qgis.org/en/site/) and further modified using Inkscape 0.91 (https://inkscape.org/en/)].

Surveys were carried out in 147 locations representing seven distinct biogeographic regions in India^56^ between November 2012 and March 2017 (Fig. 4). For the purpose of the surveys, all anurans were sampled and they are referred to, henceforth as frogs. During the surveys we selected a stream or a pond, and all frogs were captured *ad libitum* from 18:00 h to 24:00 h. Surveys targeted the two monsoons: southwest monsoon (between June and September), and northeast monsoon (between October and November). Sites that exceeded 600 m above sea level were selected in the Indian mainland, as the sites had optimal abiotic conditions for survival and growth of *Bd*. Each frog was hand caught using a pair of fresh non-powdered gloves and held in afresh plastic bag to eliminate cross-contamination. We captured at least 10 different frogs, without any preference for each species at any given site. Swab samples were collected from frogs by swabbing the skin using sterile cotton swabs (HIMEDIA^®^PW003). Each swab was stroked 70 times on the frog as follows:10 strokes each onthe dorsal surface, lateral sides, ventral surface and undersides of the thighs and five outward strokes on the undersides of each foot^57^. After swabbing each individual was examined for clinical symptoms such as loss of righting reflex, abnormal body posture, skin sloughing, skin lesions, and other abnormalities, after which, the frog was released at the site of capture. Geo-coordinates and altitude were measured using the handheld GPS, Garmin eTrex 10. Used gloves and plastic bags were incinerated at the end of the sampling session. Instruments and boots were cleaned with bleach and wiped with ethanol before proceeding to the next sampling location. All methods were carried out in accordance with Florida International University’s Institutional Animal Care and use Committee Guidelines (IACUC: 12-004-CR-A3096-01 and 13-034-CR01-200222). Work permits were obtained from Andaman and Nicobar Forest Department (CWLW/WL/134B/399), Tamil Nadu Forest Department (WL5-A/002322/2016), Kerala Forest Department (WL10-8538/2015), and Arunachal Pradesh Forest Department (CWL/G/13(17)/06-07/PT-III/4306-13) to carry out the fieldwork and to collect swab samples.

### DNA extraction

DNA was extracted from the cotton swabs as per the protocol devised by Goka *et al*.^8^ with some modifications. Swabs were cut and placed into 2 ml tubes containing 300 µl of lysis buffer [See lysis buffer composition Goka *et al*.^8^] along with three tungsten carbide beads (3 mm diameter) and were homogenised in Qiagen Tissue Lyser II for 2 min followed by centrifugation at 8,000 rpm for 2 min. The supernatant containing DNA was transferred into fresh 1.5 ml tubes and incubated initially at 50 °C for 2 h, followed by incubation at 95°C for 20 min. After incubation, these tubes were centrifuged at 13,000 rpm for 10 min and the supernatant was transferred into fresh 1.5 ml tubes and stored at −20 °C. This DNA extract was diluted with nuclease-free water in the proportions of 1:10 to be used as a template for PCR analysis.

### *Bd* detection and haplotyping

Initially, we tested the efficiency of two assays for detecting *Bd* from our samples. For this, we used both Quantitative PCR (qPCR) and Nested PCR on roughly 50% of our samples. qPCR was carried out using the protocol from Kriger *et al*.^58^ with a reaction volume of 10 µl. containing 1X Takara master mix (premix Ex Taq), 900 nM of forward, and reverse primer (ITS1-3 Chytr, 5.8S Chytr), 250 nM Taqman MGB probe, 1X ROX (TAKARA Bio.Inc.), 2 µl of diluted DNA, and the remaining volume made up by nuclease-free water. Cycle conditions for qPCR were as follows: initial denaturation at 95 °C for 10 min, followed by 50 cycles of denaturation at 95 °C for 15 sec; annealing, and extension at 60 °C, for 1 min. We used qPCR standards at 100, 10, 1 and 0.1 Genome Equivalents (GE). All reactions were carried out in duplicates with positive (*Bd *JEL 423 DNA) and negative control (nuclease-free water). For nested PCRs, the ITS1-5.8S-ITS2 region (~300 bp) was used as the marker. We followed Goka *et al*.^8^ nested PCR protocol and performed the first PCR in a total 10 µl reaction containing 1X of Emerald Takara master mix, 1 picomole each of primers Bd18SF, and Bd28SR1^8^, 1 µl of 1:10 diluted template DNA and the remaining volume was made up by adding molecular grade water. The PCR conditions were as follows: initial denaturation at 95 °C for 10 min, followed by 40 cycles of denaturation at 95 °C for 30 sec, primer annealing at 50 °C for 30 sec, extension at 72 °C for 30 sec, and a final extension at 72 °C for 10 min. We included a positive control (*Bd *JEL 423 DNA), and a negative control (nuclease-free water) in every run. For the second PCR, we used Bd 1a and Bd 2b primers^8,59^, and 1 µl of first PCR product as DNA template for second PCR. PCR conditions as follows: initial denaturation at 95 °C for 10 min, followed by 30 cycles of denaturation at 95 °C for 30 sec, primer annealing at 65 °C for 30 sec, extension at 72 °C for 30 sec, and a final extension at 72 °C for 10 min. PCR products were visualised on 2% agarose gels, 300 bp bands were observed, and samples positive for *Bd* were re-amplified. Sequencing was done twice using Applied Biosystems (model 3730).

Few samples could not be reliably sequenced due to multiple *Bd* ITS DNA strands in a single sample, so we created clones in *Escherichia coli* to address this problem. For this, primers Bda (5′-CAGTGTGCCATATGTCACG-3′) and Bdb (5′-CATGGTTCATATCTGTCCAG-3′) were modified by inserting the restriction sites for enzymes EcoRI and PstI at 5′- end of the primers. The resultant primers BdaP (5′-CACTGCAGTGTGCCATATGTCACG-3′) and BdbE (5′-GGAATTCCCATGGTTCATATCTGTCCAG-3′) were used for amplification of the 300 bp fragment. The PCR conditions used for amplification were: initial denaturation at 95 °C for 5 min, 30 cycles of amplification with each cycle containing 95 °C for 15 sec, 65 °C for 30 sec, 72 °C for 30 sec, and a final extension at 72 °C for 5 min. The amplicons were gel purified, digested with the enzymes EcoRI, and PstI, and after digestion they were re-purified by gel elution. The purified amplicons were cloned by ligating into a pBS-SK vector that was linearized with EcoRI and PstI. Ligated products were transformed into *E. coli* DH5α bacterial cells that were made competent. White transformants were selected on LB agar plates containing ampicillin (60 µg/ml) and X-gal (5-bromo-4-chloro-3-indolyl-β-D-galactoside; 20 µg/ml). Colony PCR was performed with the transformants using the M13 forward, and reverse primers. The amplicons obtained were then sequenced with the M13 primer.

### Data analyses

For estimating prevalence, we used ‘binconf’^60–62^ function with Wilson score interval in the Hmisc package in R^63^ (version 3.4.1) to estimate a 95% confidence interval (CI). We used Estimate S (version 9.1) to: (i) obtain second-order Jack-knife extrapolation estimate^64^ to arrive at the number *Bd* haplotypes that could be present in the samples, as there is expected to be an incomplete detection of rare haplotypes in the samples^65^; (ii) to check for differences in haplotype richness in different geographic regions by using rarefaction, for the smallest sample (N = 50) in any particular region. Samples from the North-East hills were excluded from this analysis, as there were no positives. One-way analysis of variance (ANOVA) was performed to test for differences in *Bd* prevalence in the geographic regions, using R (version 3.4.1). A Bayesian tree was prepared using *Bd* ITS1-5.8S-ITS2 sequences available at genebank, and the sequences generated in this study. The species, *Terramyces subangulosm*, *Boothiomyces* sp., *Boothiomyces macroporosum*, and *Kappamyces laurelensis* were used as outgroups (Supplementary Table S3). We used, Clustal W^66^ in MEGA^67^ (version 6.06) to align all the sequences and prepared a nexus file. JModel test was performed to obtainthe best evolutionary model using JModel^68^ (version 2.1.10) using Bayesian Information Criteria (BIC). The HKY + G evolutionary model was identified as the best model. We used FastGap^69^ (version 1.2) to create a gap matrix. A Bayesian tree was constructed using the new matrix obtained in MrBayes^70,71^ (version 3.2.6) with HKY + G evolutionary model. Fifteen million generations were chosen, and sampling was done after every 500 generations. To ensure effective sampling size, Tracer (version 1.6) was used. After the initial 25% of trees were discarded as burn-ins, the resultant tree was visualised in FigTree (version 1.4.2). All *Bd* ITS sequences, except the outgroup, were aligned using ClustalW^66^ in MEGA^67^ (version6.06) with some manual editing. A FASTA file was generated through MEGA^67^ (version 6.06), and it was used in DnaSP^72^(version 5) to count the number of haplotypes and prepare Roehl data file. Network(version 5.0; http://www.fluxus-engineering.com) was used to build haplotype network using median-joining method^73^.

### Data Accessibility

DNA sequences were submitted at Genbank (accession no. MG252074 - MG252130).

## Electronic supplementary material

Supplementary information
