# Supplementary material for: Enzootic frog pathogen Batrachochytrium dendrobatidis in Asian tropics reveals high ITS haplotype diversity and low prevalence
Source: Sci Rep. 2018 Jul 4;8:10125. doi: 10.1038/s41598-018-28304-1 (PMC6031667; doi:10.1038/s41598-018-28304-1)
Supplement: Supplementary file 1 — Supplementary information [file 41598_2018_28304_MOESM1_ESM.docx]

**Enzootic frog pathogen *Batrachochytrium dendrobatidis* in Asian tropics reveals high ITS haplotype diversity and low prevalence**

**AUTHORS**:

Milind C. Mutnale^1^, Sachin Anand^1^, Lilly M. Eluvathingal^2^, Jayanta K. Roy^3^, Gundlapally S. Reddy^1^, Karthikeyan Vasudevan^1*^

**AUTHORS AFFILIATIONS:**

1. CSIR-Centre for Cellular and Molecular Biology, Laboratory for the Conservation of Endangered Species, Hyderabad, Telangana, India.

2. Biology Department, Occidental College, Los Angeles, California, 90041

3. Department of Life Science and Bioinformatics, Assam University, Diphu Campus, Karbi Anglong–782460, Assam, India.

***Corresponding Author:**

Karthikeyan Vasudevan, CSIR-Centre for Cellular and Molecular Biology, Laboratory for the Conservation of Endangered Species, Hyderabad, Telangana, India. [karthik@ccmb.res.in](mailto:karthik@ccmb.res.in)

Supplementary Table S1. *Bd* ITS1-5.8S-ITS2 sequences included in our Taqman binding site mutation analyses.

| **Haplotype** | **Accession number** | **Reference** |
| --- | --- | --- |
| BR11 | JQ582896 | Schloegel et al. 2012 |
| BR15 | JQ582914 | Schloegel et al. 2012 |
| BR21 | JQ582932 | Schloegel et al. 2012 |
| JP02 | AB435212 | Goka et al. 2009 |
| JP07 | AB435217 | Goka et al. 2009 |
| JP09 | AB435219 | Goka et al. 2009 |
| JP10 | AB435220 | Goka et al. 2009 |
| JP12 | AB435222 | Goka et al. 2009 |
| JP13 | AB435223 | Goka et al. 2009 |
| JP38 | AB723974 | Goka et al. unpublished |
| JP39 | AB723975 | Goka et al. unpublished |
| JP42 | AB723978 | Goka et al. unpublished |
| JP45 | AB723981 | Goka et al. unpublished |
| JP52 | AB724254 | Goka et al. unpublished |
| JP54 | AB733410 | Goka et al. unpublished |
| JP58 | AB724259 | Goka et al. unpublished |
| CN07 | JN870747 | Bai et al. 2012 |
| CN24 | JN870760 | Bai et al. 2012 |
| CN30 | JN870752 | Bai et al. 2012 |
| KR12 | JX983052 | Bataille et al. 2013 |
| KR15 | JX983055 | Bataille et al. 2013 |
| KR16 | JX983056 | Bataille et al. 2013 |
| KR22 | JX983061 | Bataille et al. 2013 |
| KR23 | JX983062 | Bataille et al. 2013 |
| KR24 | JX983063 | Bataille et al. 2013 |
| KR25 | JX983064 | Bataille et al. 2013 |
| KR40 | JX983079 | Bataille et al. 2013 |
| KR41 | JX983080 | Bataille et al. 2013 |
| KR43 | JX983082 | Bataille et al. 2013 |
| IN05 | MG252078 | Present study |
| IN17 | MG252090 | Present study |
| IN31 | MG252104 | Present study |
| IN55 | MG252128 | Present study |

Supplementary Table S2. *Bd* ITS1-5.8S-ITS2 sequences included in our qPCR reverse primer.

| **Haplotype** | **Accession number** | **Reference** |
| --- | --- | --- |
| JP55 | AB469198 | Goka et al. unpublished |
| CN18 | JN870759 | Bai et al. 2012 |
| CN25 | JN870757 | Bai et al. 2012 |
| CN26 | JN870761 | Bai et al. 2012 |
| CN27 | JN870758 | Bai et al. 2012 |
| KR33 | JX983072 | Bataille et al. 2013 |
| KR39 | JX983078 | Bataille et al. 2013 |
| KR40 | JX983079 | Bataille et al. 2013 |
| KR47 | JX983086 | Bataille et al. 2013 |
| KR48 | JX983087 | Bataille et al. 2013 |
| KR50 | JX983089 | Bataille et al. 2013 |
| IN16 | MG252089 | Present study |
| IN17 | MG252090 | Present study |
| IN38 | MG252111 | Present study |
| IN42 | MG252115 | Present study |
| IN49 | MG252122 | Present study |
| IN57 | MG252130 | Present study |

Supplementary Table S3. *Bd* ITS1-5.8S-ITS2 sequences included in our Bayesian phylogenetic analyses.

| **Haplotype** | **Identification** | **Accession number** | | **Reference** |  |
| --- | --- | --- | --- | --- | --- |
| BR03 | UM142 E | JQ582886 | Schloegel et al. 2012 | | |
| BR04 | UM142 H | JQ582887 | Schloegel et al. 2012 | | |
| BR05 | JEL 648 B | JQ582889 | Schloegel et al. 2012 | | |
| BR06 | JEL 648 N | JQ582890 | Schloegel et al. 2012 | | |
| BR07 | JEL 648 E | JQ582891 | Schloegel et al. 2012 | | |
| BR08 | JEL 648 F | JQ582892 | Schloegel et al. 2012 | | |
| BR09 | JEL 648 K | JQ582894 | Schloegel et al. 2012 | | |
| BR10 | JEL 648 G | JQ582919 | Schloegel et al. 2012 | | |
| BR11 | UM142 H | JQ582896 | Schloegel et al. 2012 | | |
| BR12 | UM142 G | JQ582898 | Schloegel et al. 2012 | | |
| BR13 | UM142 P | JQ582900 | Schloegel et al. 2012 | | |
| BR14 | JEL 648 C | JQ582902 | Schloegel et al. 2012 | | |
| BR15 | JEL 648 H | JQ582914 | Schloegel et al. 2012 | | |
| BR16 | UM142 R | JQ582908 | Schloegel et al. 2012 | | |
| BR17 | UM142 N | JQ582909 | Schloegel et al. 2012 | | |
| BR18 | UM142 S | JQ582913 | Schloegel et al. 2012 | | |
| BR19 | JEL 648 J | JQ582917 | Schloegel et al. 2012 | | |
| BR20 | UM142 I | JQ582930 | Schloegel et al. 2012 | | |
| BR21 | UM142 Q | JQ582932 | Schloegel et al. 2012 | | |
| BR22 | JEL 648 M | JQ582933 | Schloegel et al. 2012 | | |
| BR23 | UM142 L | JQ582939 | Schloegel et al. 2012 | | |
| BR24 | UM142 J | JQ582934 | Schloegel et al. 2012 | | |
| BR25 | UM142 D | JQ582936 | Schloegel et al. 2012 | | |
| SA01 | CW 34 Q | JQ582905 | Schloegel et al. 2012 | | |
| SA02 | CW 34 L | JQ582916 | Schloegel et al. 2012 | | |
| SA03 | CW 34 R | JQ582921 | Schloegel et al. 2012 | | |
| SA04 | CW 34 K | JQ582935 | Schloegel et al. 2012 | | |
| SA05 | CW 34 M | JQ582940 | Schloegel et al. 2012 | | |
| SA06 | CW 34 F | JQ582927 | Schloegel et al. 2012 | | |
| SA07 | CW 34 N | JQ582929 | Schloegel et al. 2012 | | |
| SA08 | CW 34 T | JQ582937 | Schloegel et al. 2012 | | |
| SA09 | CW 34 I | JQ582938 | Schloegel et al. 2012 | | |
| SA10 | CW 34 H | JQ582941 | Schloegel et al. 2012 | | |
| SA11 | CW 34 D | JQ582906 | Schloegel et al. 2012 | | |
| TE01 | TK01 | EU779861 | Gaertner et al. unpublished | | |
| TE02 | NE14 | EU779863 | Gaertner et al. unpublished | | |
| TE05 | DA03_Bd_JG05_06 | FJ373880 | Gaertner et al. unpublished | | |
| TE06 | AD27_Bd_JG77_78 | FJ373881 | Gaertner et al. unpublished | | |
| TE07 | DA04_Bd_JG07_08 | FJ373882 | Gaertner et al. unpublished | | |
| JP01 | BD 01 | AB435211 | Goka et al. 2009 | | |
| JP02 | BD 02 | AB435212 | Goka et al. 2009 | | |
| JP03 | BD 03 | AB435213 | Goka et al. 2009 | | |
| JP04 | BD 04 | AB435214 | Goka et al. 2009 | | |
| JP05 | BD 05 | AB435215 | Goka et al. 2009 | | |
| JP06 | BD 06 | AB435216 | Goka et al. 2009 | | |
| JP07 | BD 07 | AB435217 | Goka et al. 2009 | | |
| JP08 | BD 08 | AB435218 | Goka et al. 2009 | | |
| JP09 | BD 09 | AB435219 | Goka et al. 2009 | | |
| JP10 | BD 10 | AB435220 | Goka et al. 2009 | | |
| JP11 | BD 11 | AB435221 | Goka et al. 2009 | | |
| JP12 | BD 12 | AB435222 | Goka et al. 2009 | | |
| JP13 | BD 13 | AB435223 | Goka et al. 2009 | | |
| JP14 | BD 14 | AB435224 | Goka et al. 2009 | | |
| JP15 | BD 15 | AB435225 | Goka et al. 2009 | | |
| JP16 | BD 16 | AB435226 | Goka et al. 2009 | | |
| JP17 | BD 17 | AB435227 | Goka et al. 2009 | | |
| JP18 | BD 18 | AB435228 | Goka et al. 2009 | | |
| JP19 | BD 19 | AB435229 | Goka et al. 2009 | | |
| JP20 | BD 20 | AB435230 | Goka et al. 2009 | | |
| JP21 | BD 21 | AB435231 | Goka et al. 2009 | | |
| JP22 | BD 22 | AB469195 | Goka et al. 2009 | | |
| JP23 | BD 23 | AB469196 | Goka et al. 2009 | | |
| JP24 | BD 24 | AB469197 | Goka et al. 2009 | | |
| JP25 | BD 25 | AB469198 | Goka et al. 2009 | | |
| JP26 | BD 26 | AB469199 | Goka et al. 2009 | | |
| CN01 | CN1 | JN870740 | Bai et al. 2012 | | |
| CN02 | CN2 | JN870741 | Bai et al. 2012 | | |
| CN03 | CN3 | JN870755 | Bai et al. 2012 | | |
| CN04 | CN4 | JN870744 | Bai et al. 2012 | | |
| CN05 | CN5 | JN870742 | Bai et al. 2012 | | |
| CN06 | CN6 | JN870756 | Bai et al. 2012 | | |
| CN07 | CN7 | JN870747 | Bai et al. 2012 | | |
| CN08 | CN8 | JN870749 | Bai et al. 2012 | | |
| CN09 | CN9 | JN870750 | Bai et al. 2012 | | |
| CN10 | CN10 | JN870753 | Bai et al. 2012 | | |
| CN11 | CN11 | JN870754 | Bai et al. 2012 | | |
| CN12 | CN12 | JN870743 | Bai et al. 2012 | | |
| CN13 | CN13 | JN870768 | Bai et al. 2012 | | |
| CN14 | CN14 | JN870769 | Bai et al. 2012 | | |
| CN15 | CN15 | JN870767 | Bai et al. 2012 | | |
| CN16 | CN16 | JN870745 | Bai et al. 2012 | | |
| CN17 | CN17 | JN870746 | Bai et al. 2012 | | |
| CN18 | CN18 | JN870759 | Bai et al. 2012 | | |
| CN19 | CN19 | JN870762 | Bai et al. 2012 | | |
| CN20 | CN20 | JN870766 | Bai et al. 2012 | | |
| CN21 | CN21 | JN870765 | Bai et al. 2012 | | |
| CN22 | CN22 | JN870763 | Bai et al. 2012 | | |
| CN23 | CN23 | JN870764 | Bai et al. 2012 | | |
| CN24 | CN24 | JN870760 | Bai et al. 2012 | | |
| CN25 | CN25 | JN870757 | Bai et al. 2012 | | |
| CN26 | CN26 | JN870761 | Bai et al. 2012 | | |
| CN27 | CN27 | JN870758 | Bai et al. 2012 | | |
| CN28 | CN28 | JN870751 | Bai et al. 2012 | | |
| CN29 | CN29 | JN870748 | Bai et al. 2012 | | |
| CN30 | CN30 | JN870752 | Bai et al. 2012 | | |
| KR02 | KR2 | JX983045 | Bataille et al. 2013 | | |
| KR04 | KR4 | JX983046 | Bataille et al. 2013 | | |
| KR05 | KR5 | JX983047 | Bataille et al. 2013 | | |
| KR08 | KR8 | JX983048 | Bataille et al. 2013 | | |
| KR09 | KR9 | JX983049 | Bataille et al. 2013 | | |
| KR10 | KR10 | JX983050 | Bataille et al. 2013 | | |
| KR11 | KR11 | JX983051 | Bataille et al. 2013 | | |
| KR12 | KR12 | JX983052 | Bataille et al. 2013 | | |
| KR13 | KR13 | JX983053 | Bataille et al. 2013 | | |
| KR14 | KR14 | JX983054 | Bataille et al. 2013 | | |
| KR15 | KR15 | JX983055 | Bataille et al. 2013 | | |
| KR16 | KR16 | JX983056 | Bataille et al. 2013 | | |
| KR17 | KR17 | JX983057 | Bataille et al. 2013 | | |
| KR19 | KR19 | JX983058 | Bataille et al. 2013 | | |
| KR20 | KR20 | JX983059 | Bataille et al. 2013 | | |
| KR21 | KR21 | JX983060 | Bataille et al. 2013 | | |
| KR22 | KR22 | JX983061 | Bataille et al. 2013 | | |
| KR23 | KR23 | JX983062 | Bataille et al. 2013 | | |
| KR24 | KR24 | JX983063 | Bataille et al. 2013 | | |
| KR25 | KR25 | JX983064 | Bataille et al. 2013 | | |
| KR26 | KR26 | JX983065 | Bataille et al. 2013 | | |
| KR27 | KR27 | JX983066 | Bataille et al. 2013 | | |
| KR28 | KR28 | JX983067 | Bataille et al. 2013 | | |
| KR29 | KR29 | JX983068 | Bataille et al. 2013 | | |
| KR30 | KR30 | JX983069 | Bataille et al. 2013 | | |
| KR31 | KR31 | JX983070 | Bataille et al. 2013 | | |
| KR32 | KR32 | JX983071 | Bataille et al. 2013 | | |
| KR33 | KR33 | JX983072 | Bataille et al. 2013 | | |
| KR34 | KR34 | JX983073 | Bataille et al. 2013 | | |
| KR35 | KR35 | JX983074 | Bataille et al. 2013 | | |
| KR36 | KR36 | JX983075 | Bataille et al. 2013 | | |
| KR37 | KR37 | JX983076 | Bataille et al. 2013 | | |
| KR38 | KR38 | JX983077 | Bataille et al. 2013 | | |
| KR39 | KR39 | JX983078 | Bataille et al. 2013 | | |
| KR40 | KR40 | JX983079 | Bataille et al. 2013 | | |
| KR41 | KR41 | JX983080 | Bataille et al. 2013 | | |
| KR42 | KR42 | JX983081 | Bataille et al. 2013 | | |
| KR43 | KR43 | JX983082 | Bataille et al. 2013 | | |
| KR44 | KR44 | JX983083 | Bataille et al. 2013 | | |
| KR45 | KR45 | JX983084 | Bataille et al. 2013 | | |
| KR46 | KR46 | JX983085 | Bataille et al. 2013 | | |
| KR47 | KR47 | JX983086 | Bataille et al. 2013 | | |
| KR48 | KR48 | JX983087 | Bataille et al. 2013 | | |
| KR49 | KR49 | JX983088 | Bataille et al. 2013 | | |
| KR50 | KR50 | JX983089 | Bataille et al. 2013 | | |
| IT01 | MIB:zpl 00671 | FJ010547 | Federici et al. 2008 | | |
| IN01 | IN01 | MG252074 | Present study | | |
| IN02 | IN02 | MG252075 | Present study | | |
| IN03 | IN03 | MG252076 | Present study | | |
| IN04 | IN04 | MG252077 | Present study | | |
| IN05 | IN05 | MG252078 | Present study | | |
| IN06 | IN06 | MG252079 | Present study | | |
| IN07 | IN07 | MG252080 | Present study | | |
| IN08 | IN08 | MG252081 | Present study | | |
| IN09 | IN09 | MG252082 | Present study | | |
| IN10 | IN10 | MG252083 | Present study | | |
| IN11 | IN11 | MG252084 | Present study | | |
| IN12 | IN12 | MG252085 | Present study | | |
| IN13 | IN13 | MG252086 | Present study | | |
| IN14 | IN14 | MG252087 | Present study | | |
| IN15 | IN15 | MG252088 | Present study | | |
| IN16 | IN16 | MG252089 | Present study | | |
| IN17 | IN17 | MG252090 | Present study | | |
| IN18 | IN18 | MG252091 | Present study | | |
| IN19 | IN19 | MG252092 | Present study | | |
| IN20 | IN20 | MG252093 | Present study | | |
| IN21 | IN21 | MG252094 | Present study | | |
| IN22 | IN22 | MG252095 | Present study | | |
| IN23 | IN23 | MG252096 | Present study | | |
| IN24 | IN24 | MG252097 | Present study | | |
| IN25 | IN25 | MG252098 | Present study | | |
| IN26 | IN26 | MG252099 | Present study | | |
| IN27 | IN27 | MG252100 | Present study | | |
| IN28 | IN28 | MG252101 | Present study | | |
| IN29 | IN29 | MG252102 | Present study | | |
| IN30 | IN30 | MG252103 | Present study | | |
| IN31 | IN31 | MG252104 | Present study | | |
| IN32 | IN32 | MG252105 | Present study | | |
| IN33 | IN33 | MG252106 | Present study | | |
| IN34 | IN34 | MG252107 | Present study | | |
| IN35 | IN35 | MG252108 | Present study | | |
| IN36 | IN36 | MG252109 | Present study | | |
| IN37 | IN37 | MG252110 | Present study | | |
| IN38 | IN38 | MG252111 | Present study | | |
| IN39 | IN39 | MG252112 | Present study | | |
| IN40 | IN40 | MG252113 | Present study | | |
| IN41 | IN41 | MG252114 | Present study | | |
| IN42 | IN42 | MG252115 | Present study | | |
| IN43 | IN43 | MG252116 | Present study | | |
| IN44 | IN44 | MG252117 | Present study | | |
| IN45 | IN45 | MG252118 | Present study | | |
| IN46 | IN46 | MG252119 | Present study | | |
| IN47 | IN47 | MG252120 | Present study | | |
| IN48 | IN48 | MG252121 | Present study | | |
| IN49 | IN49 | MG252122 | Present study | | |
| IN50 | IN50 | MG252123 | Present study | | |
| IN51 | IN51 | MG252124 | Present study | | |
| IN52 | IN52 | MG252125 | Present study | | |
| IN53 | IN53 | MG252126 | Present study | | |
| IN54 | IN54 | MG252127 | Present study | | |
| IN55 | IN55 | MG252128 | Present study | | |
| IN56 | IN56 | MG252129 | Present study | | |
| IN57 | IN57 | MG252130 | Present study | | |
| *K.laurelensis* |  | DQ485666 | Letcher et al. 2006 | | |
| *B.macroporosum* |  | DQ485642 | Letcher et al. 2006 | | |
| *B.macroporosum* |  | AY997084 | James et al. 2006 | | |
| *Boothiomyces sp* | | EF585656 | Letcher et al. 2008 | | |
| *T. subangulosum* | | NR119592 | Letcher et al. 2006 | | |

Supplementary Table S4. Percentage of each haplotype of *Bd* at different locations in India.

| **Region** | **Location** | **Haplotype No.** | **Frequency** | **Percentage (%)** |
| --- | --- | --- | --- | --- |
| **Western Ghats** | KMTR | IN02 | 18 | 45 |
|  |  | IN10 | 3 | 7.5 |
|  |  | IN03 | 3 | 7.5 |
|  |  | IN08 | 2 | 5 |
|  |  | IN06 | 2 | 5 |
|  |  | IN18 | 1 | 2.5 |
|  |  | IN17 | 1 | 2.5 |
|  |  | IN16 | 1 | 2.5 |
|  |  | IN15 | 1 | 2.5 |
|  |  | IN14 | 1 | 2.5 |
|  |  | IN13 | 1 | 2.5 |
|  |  | IN12 | 1 | 2.5 |
|  |  | IN11 | 1 | 2.5 |
|  |  | IN09 | 1 | 2.5 |
|  |  | IN05 | 1 | 2.5 |
|  |  | IN04 | 1 | 2.5 |
|  |  | IN01 | 1 | 2.5 |
|  |  |  |  |  |
|  | Srivillipathur | IN02 | 5 | 71.4 |
|  |  | IN10 | 1 | 14.3 |
|  |  | IN14 | 1 | 14.3 |
|  |  |  |  |  |
|  | Munnar | IN02 | 13 | 39.4 |
|  |  | IN10 | 3 | 9.1 |
|  |  | IN52 | 2 | 6.1 |
|  |  | IN40 | 1 | 3.0 |
|  |  | IN41 | 1 | 3.0 |
|  |  | IN42 | 1 | 3.0 |
|  |  | IN43 | 1 | 3.0 |
|  |  | IN44 | 1 | 3.0 |
|  |  | IN45 | 1 | 3.0 |
|  |  | IN46 | 1 | 3.0 |
|  |  | IN47 | 1 | 3.0 |
|  |  | IN48 | 1 | 3.0 |
|  |  | IN49 | 1 | 3.0 |
|  |  | IN50 | 1 | 3.0 |
|  |  | IN51 | 1 | 3.0 |
|  |  | IN53 | 1 | 3.0 |
|  |  | IN54 | 1 | 3.0 |
|  |  | IN55 | 1 | 3.0 |
|  |  |  |  |  |
|  | Dharwad | IN02 | 10 | 76.9 |
|  |  | IN10 | 2 | 15.4 |
|  |  | IN39 | 1 | 7.7 |
|  |  |  |  |  |
|  | Goa | IN02 | 4 | 80 |
|  |  | IN10 | 1 | 20 |
|  |  |  |  |  |
|  | Khireshwar | IN02 | 1 | 100 |
|  |  |  |  |  |
| **Western Himalaya** | Corbette | IN02 | 2 | 33.3 |
|  |  | IN03 | 2 | 33.3 |
|  |  | IN35 | 1 | 16.7 |
|  |  | IN38 | 1 | 16.7 |
|  |  |  |  |  |
|  | Nanital | IN06 | 1 | 33.3 |
|  |  | IN36 | 1 | 33.3 |
|  |  | IN37 | 1 | 33.3 |
|  |  |  |  |  |
| **Eastern Himalaya** | Darjeeling | IN02 | 1 | 100 |
|  |  |  |  |  |
|  | Dibang Valley | IN02 | 3 | 50 |
|  |  | IN10 | 2 | 33.3 |
|  |  | IN13 | 1 | 16.7 |
|  |  |  |  |  |
| **Andaman and Nicobar Islands** | Middle Andaman | IN02 | 16 | 45.7 |
|  |  | IN10 | 6 | 17.1 |
|  |  | IN03 | 2 | 5.7 |
|  |  | IN13 | 1 | 2.9 |
|  |  | IN21 | 1 | 2.9 |
|  |  | IN22 | 1 | 2.9 |
|  |  | IN23 | 1 | 2.9 |
|  |  | IN24 | 1 | 2.9 |
|  |  | IN25 | 1 | 2.9 |
|  |  | IN26 | 1 | 2.9 |
|  |  | IN27 | 1 | 2.9 |
|  |  | IN28 | 1 | 2.9 |
|  |  | IN29 | 1 | 2.9 |
|  |  | IN30 | 1 | 2.9 |
|  |  |  |  |  |
|  | South Andaman | IN02 | 5 | 55.6 |
|  |  | IN31 | 1 | 11.1 |
|  |  | IN32 | 1 | 11.1 |
|  |  | IN33 | 1 | 11.1 |
|  |  | IN34 | 1 | 11.1 |
|  |  |  |  |  |
|  | Little Andaman | IN02 | 4 | 44.4 |
|  |  | IN03 | 1 | 11.1 |
|  |  | IN04 | 1 | 11.1 |
|  |  | IN10 | 1 | 11.1 |
|  |  | IN13 | 1 | 11.1 |
|  |  | IN20 | 1 | 11.1 |
|  |  |  |  |  |
|  | Nicobar | IN07 | 1 | 25 |
|  |  | IN19 | 1 | 25 |
|  |  | IN56 | 1 | 25 |
|  |  | IN57 | 1 | 25 |


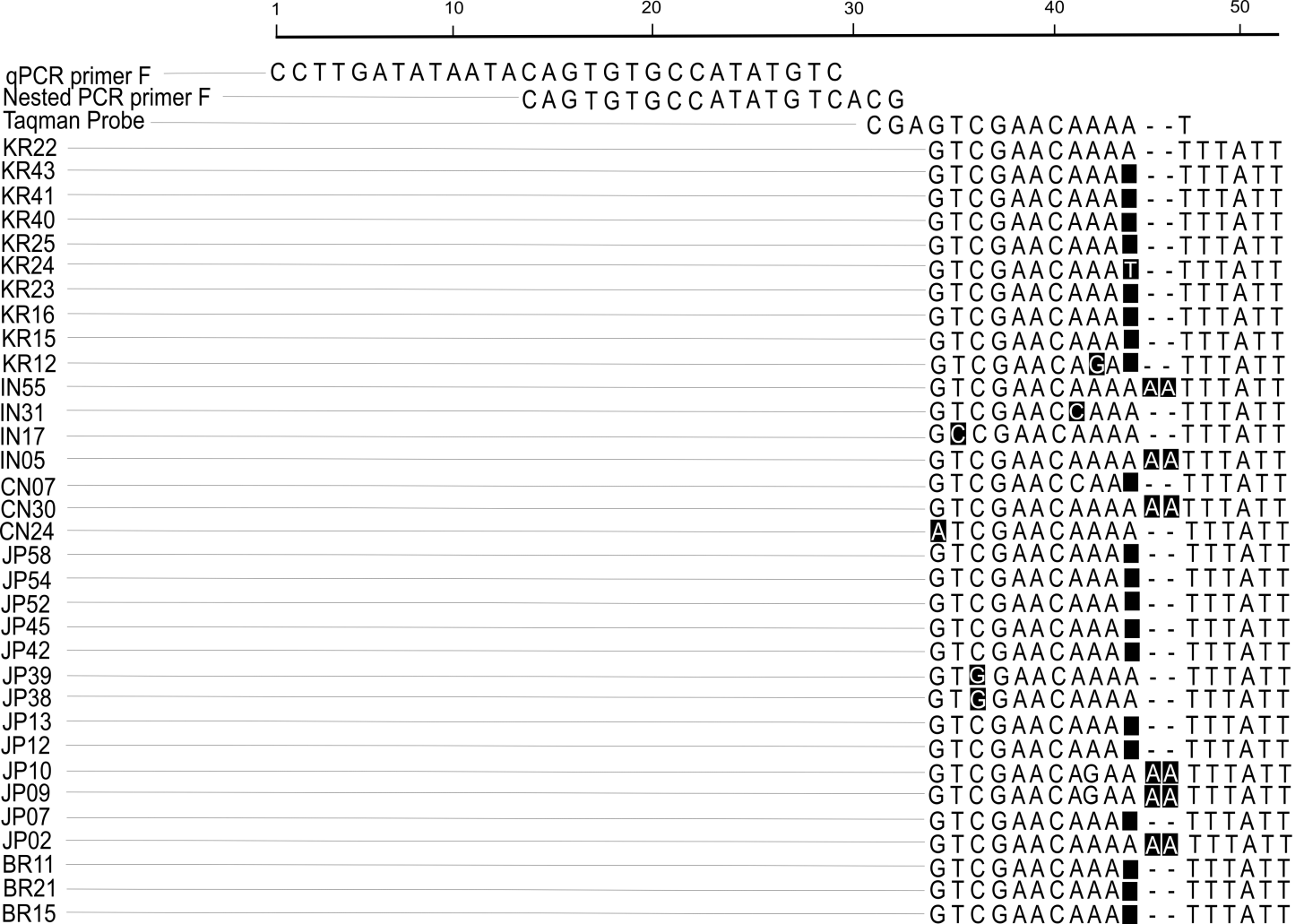


**Supplementary figure S1**. Alignment of *Bd* ITS1-5.8S-ITS2 sequences having mutations at the Taqman binding site. Sequences from haplotype KR43 to BR15 are having indels at the Taqman probe binding site (deletion of nucleotide denoted by black square and transition and transversion highlighted in black background). In total 32 sequences were found to be having this mutation from different geographic region. Abbreviations: KR-Korea, IN-India, CN-China, JP-Japan and BR-Brazil. Sequence KR22 is not having any mutation at the Taqman site. qPCR primer F is the forward primer designed for the qPCR by Boyle et al. (2004). Nested PCR primer F is forward primer designed by Annis et al. (2004) which covers two bases at the Taqman binding site.


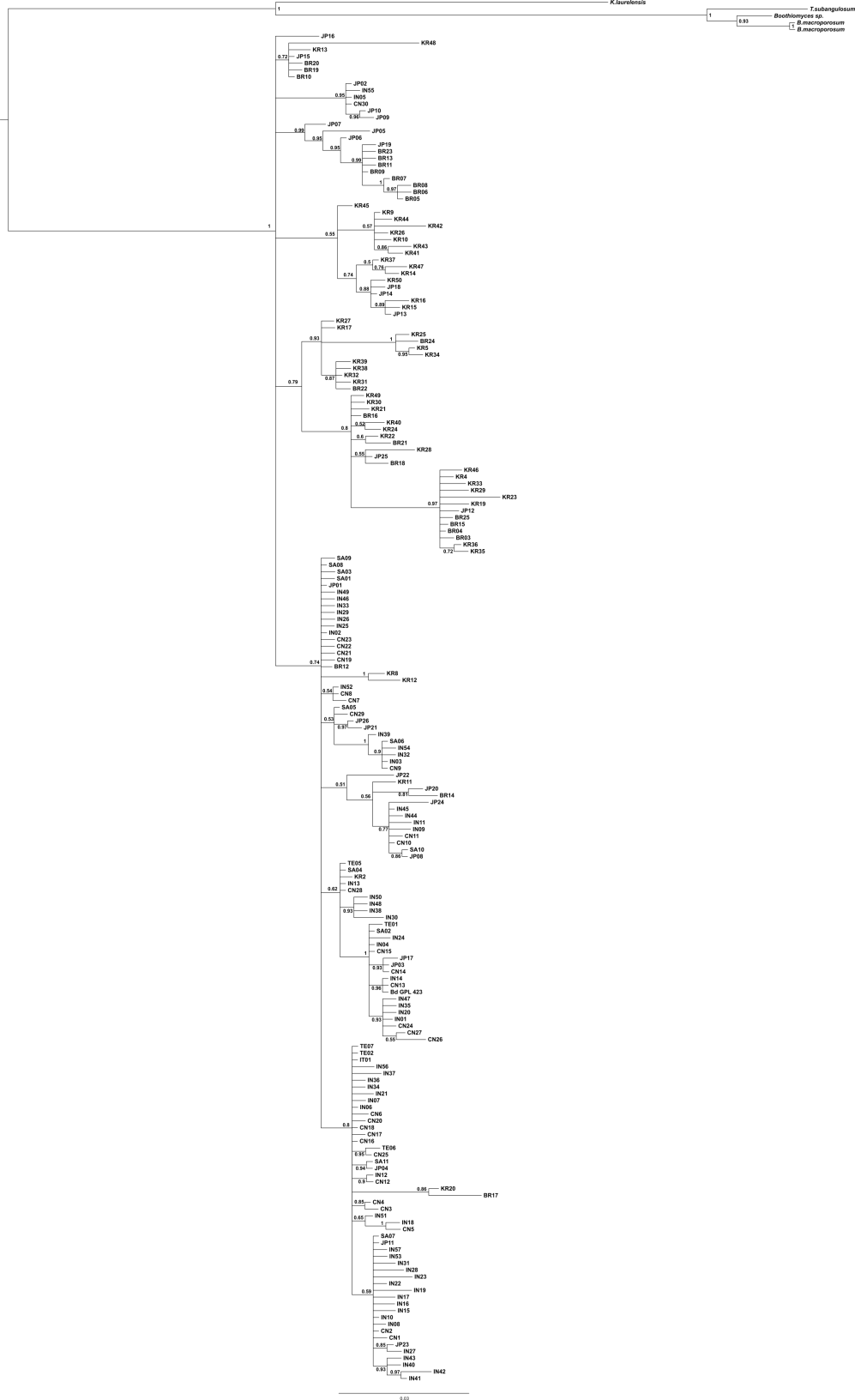


**Supplementary figure S2**. Phylogenetic tree for all *Bd* ITS haplotypes using Bayesian inference method. Number at the node represents the posterior probability (PP) that indicate the support to the clade. Only >0.5 PP have been mentioned in the tree. Outgroup sequences were used following Goka et al.(2009) and Bataille et al.(2013). Abbreviations: KR=Korea, BR=Brazil, JP=Japan, IN=India, CN=China, SA= South Africa, IT= Italy, TE= Texas. *Bd* JEL 423 ITS sequence was obtained from the www.broadinstitute.org.


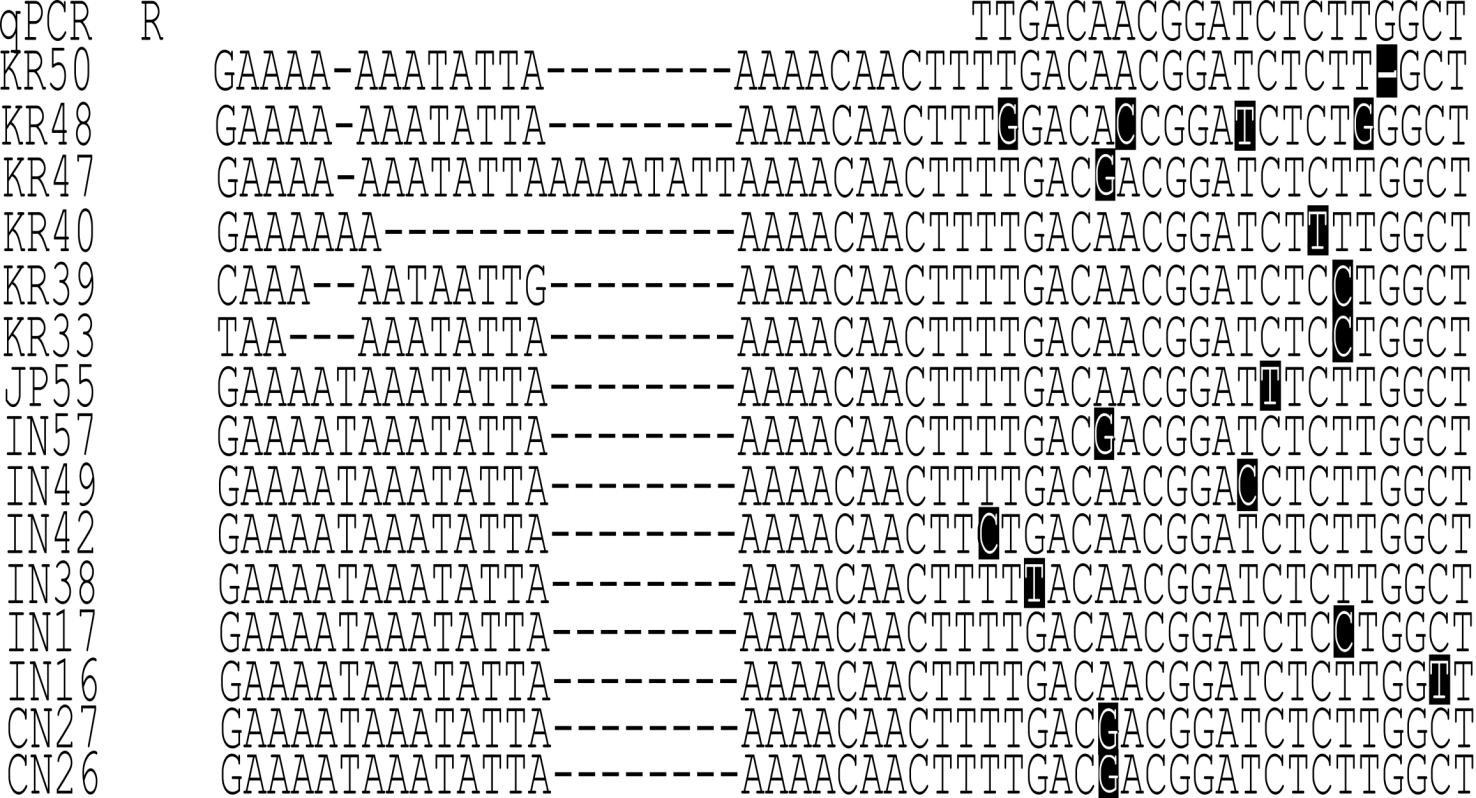


**Supplementary figure S3**. Sequences having mutations at the qPCR reverse primer site. In total 15 sequences were found to be having this mutation from different geographic region. Sequences from haplotype KR48 to CN26 consist of transition-transversion and KR50 have deletion. Abbreviations: KR-Korea, CN-China, JP-Japan, IN-India.
